# Cross-System miRNA Profiling Identifies a Clinically Recurrent miRNA Core in Colorectal Cancer

**DOI:** 10.3390/ijms27219873

**Published:** 2026-11-04

**Authors:** Deimante Valentelyte, Rokas Lukosevicius, Ruta Steponaitiene, Violeta Salteniene, Egidijus Morkunas, Zbigniev Balion, Gabija Baldauskaite, Gabija Zemgulyte, Goda Butaite, Ugne Kulokiene, Greta Gedgaudiene, Juozas Kupcinskas, Ruta Inciuraite, Jurgita Skieceviciene

**Affiliations:** 1 Institute for Digestive Research, Lithuanian University of Health Sciences, LT-50161 Kaunas, Lithuania; deimante.valentelyte@lsmu.lt (D.V.); goda.butaite@lsmu.lt (G.B.);; 2 Department of Gastroenterology, Academy of Medicine, Lithuanian University of Health Sciences, LT-50161 Kaunas, Lithuania; 3 Institute of Pharmaceutical Technologies, Faculty of Pharmacy, Lithuanian University of Health Sciences, LT-50161 Kaunas, Lithuania

**Keywords:** colorectal cancer, microRNA, extracellular vesicles, small RNA sequencing, miR-30 family, cross-system profiling

## Abstract

Colorectal cancer (CRC) encompasses biologically diverse cellular phenotypes with distinct microRNA (miRNA) expression patterns. Assessing whether cell-model-derived alterations recur in extracellular vesicles (EVs) and human tumors may help prioritize candidates with broader relevance to CRC. We performed small RNA sequencing of non-malignant HCoEpiC cells, differentiated enterocyte-like Caco-2 cells, less differentiated HCT116 cells, and their corresponding EVs. miRNAs showing concordant dysregulation in both CRC cell lines relative to HCoEpiC, with greater absolute log_2_ fold changes in HCT116, were prioritized and evaluated in human colorectal tissues and TCGA-COAD/READ. Seven of 29 prioritized cellular miRNAs met the EV selection criteria. Of the 26 candidates measurable in the patient-tissue dataset, eight showed FDR-significant, directionally concordant differences between CRC and healthy colorectal tissues, including five concordantly dysregulated across cells, EVs, and patient tissues. Analysis of 11 matched TCGA tumor–non-tumor pairs reproduced reduced miR-30a-3p, miR-30c-2-3p, and miR-1271-5p and increased miR-140-5p expression. Target-based enrichment and exploratory miRNA-mRNA correlation analyses identified associations with CRC-relevant signaling and MYC-related, cell-cycle, DNA-repair, and metabolic programs. No significant associations with the evaluated clinicopathological features or overall survival were detected. Collectively, these findings identify a reproducible subset of CRC-associated miRNA alterations and highlight reduced miR-30a-3p and miR-30c-2-3p expression as a recurrent family-level pattern warranting mechanistic investigation.

## 1. Introduction

Colorectal cancer (CRC) is characterized by molecular alterations that distinguish malignant cells from normal epithelium and contribute to differences in tumor behavior, including proliferative, invasive, and metastatic potential. CRC is a heterogeneous disease that develops through the accumulation of molecular alterations affecting epithelial homeostasis, tumor progression, and therapeutic response [1,2]. In addition to genomic and epigenetic abnormalities, post-transcriptional regulatory mechanisms also contribute to CRC heterogeneity by modulating gene expression programs involved in tumorigenesis [3,4].

Among these regulators, microRNAs (miRNAs) are of particular interest, as they can influence multiple cellular pathways involved in proliferation, apoptosis, differentiation, migration, invasion, and stress responses [5,6,7]. Several miRNAs, including miR-143/145, miR-21, miR-135a/b, and members of the miR-200 family, have been associated with CRC-relevant processes such as tumor growth, invasion, metastasis, and treatment response [5,8]. Previous studies have shown that CRC-associated miRNA signatures can distinguish malignant from non-malignant tissues and may also provide diagnostic, prognostic, or therapeutic information [5]. Identifying shared miRNA alterations across biologically distinct CRC cell models and assessing their concordance with human colorectal tissues may help prioritize candidates with broader relevance to CRC [3,6].

In addition to intracellular miRNA regulation, extracellular vesicle (EV)-associated miRNAs have attracted considerable interest in CRC because they may reflect the molecular state of donor cells, contribute to intercellular communication, and serve as potential biomarkers [7,9]. However, EV-associated miRNA cargo does not necessarily directly mirror intracellular miRNA expression, as selective miRNA sorting into EVs has been described [10,11]. Integrating cellular and EV-associated miRNA profiles may therefore help identify CRC-associated alterations that are concordant across intracellular and extracellular compartments. Nevertheless, it remains unclear which cell-model-derived miRNA alterations are reproducible across biologically distinct CRC models, their EVs, and human colorectal tumors.

Accordingly, this study aimed to identify CRC-associated miRNA alterations that recur across cellular, extracellular, and clinical datasets. miRNAs showing concordant dysregulation in two biologically distinct CRC cell lines relative to non-malignant colonic epithelial cells were prioritized. By integrating cellular and EV-associated miRNA profiles, we further assessed whether these candidates showed similar changes across intracellular and extracellular compartments. Prioritized miRNAs were subsequently evaluated in healthy colorectal (HC), adenomatous polyp (AP), and CRC tissues, followed by independent assessment in TCGA-COAD/READ. Exploratory miRNA-mRNA correlation analysis in TCGA data was used to characterize gene expression programs associated with the recurrent candidate miRNAs. This cross-system approach enabled the identification of reproducible CRC-associated miRNA alterations across experimental models and human colorectal tissues.

## 2. Results

### 2.1. Cellular miRNA Profiling Reveals Shared Alterations in CRC Cell Lines

**Figure 1:**
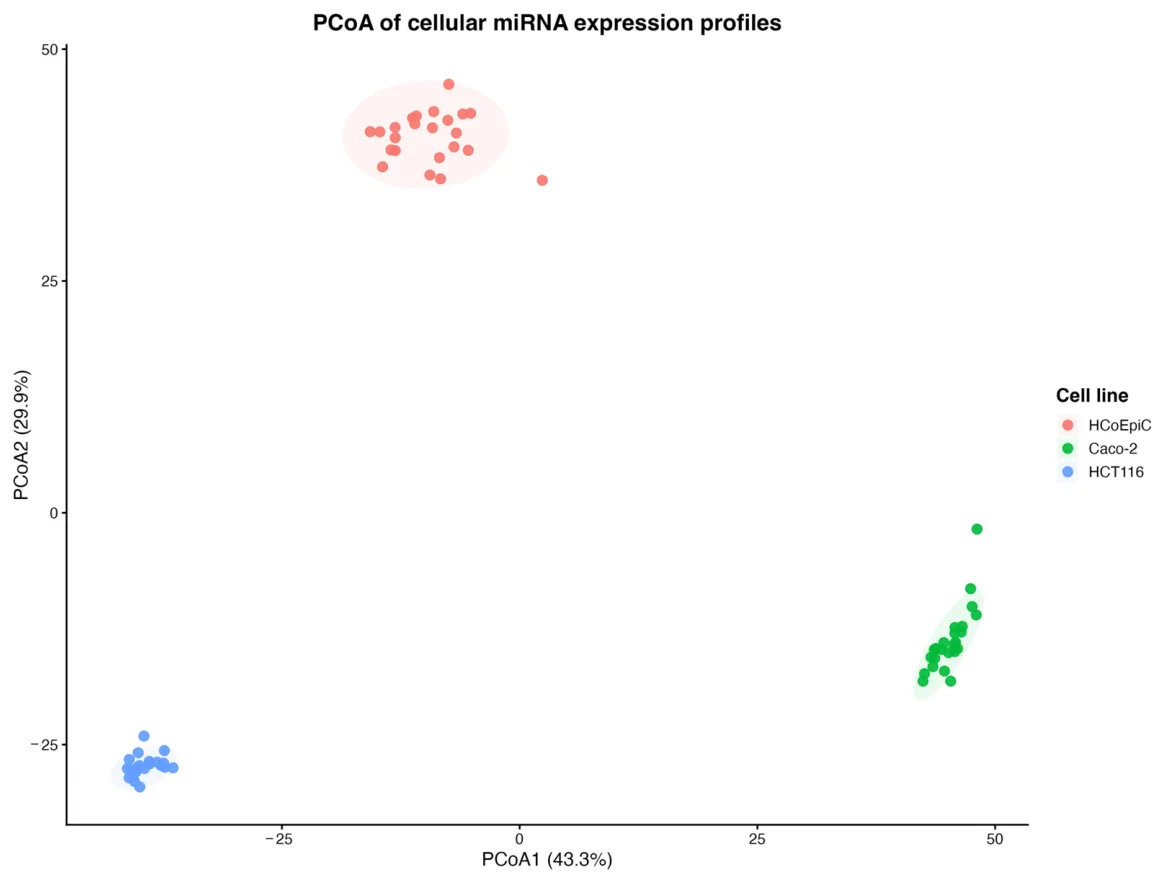
Principal coordinate analysis of cellular miRNA expression profiles. PCoA was performed using Euclidean distances calculated from variance-stabilized expression values of all 852 miRNAs retained after quality control and filtering. Each point represents one sample, and colors indicate cell line identity. Shaded areas represent 95% confidence ellipses for each cell line. The percentages shown on the axes indicate the proportion of total variation explained by the first two principal coordinates.

To characterize miRNA expression differences between normal colonic epithelial cells and CRC cell lines, small RNA sequencing data from HCoEpiC, Caco-2, and HCT116 cells were analyzed. After quality control and filtering, 852 miRNAs were retained in the cellular dataset. PCoA based on all retained miRNAs revealed a clear separation of HCoEpiC cells from both CRC cell lines, indicating distinct global miRNA expression profiles between normal and cancer-derived cells. In addition, Caco-2 and HCT116 samples formed distinct clusters, suggesting differences in miRNA expression between the two CRC models. The first two principal coordinates explained 43.3% and 29.9% of the total variation, respectively, together accounting for 73.2% of the variability in the cellular dataset (Figure 1). A corresponding PCoA of EV-associated miRNA profiles was performed to evaluate sample distribution and clustering within the EV dataset and is presented in Supplementary Figure S6.

**Figure 2:**
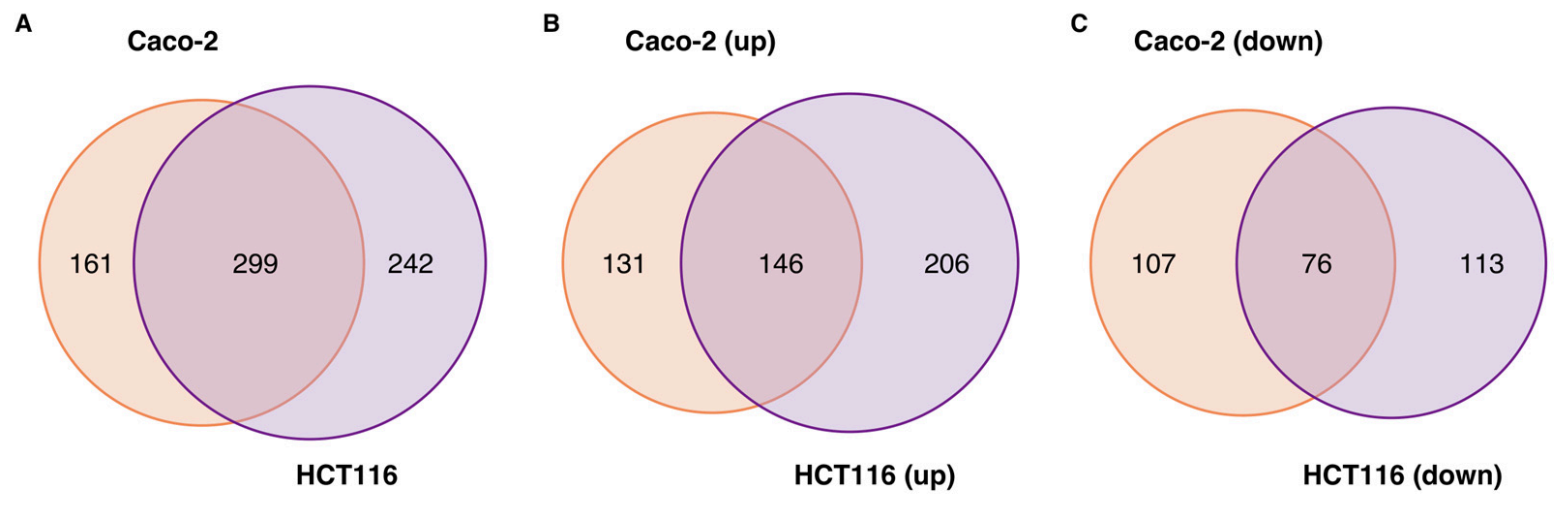
Overlap of differentially expressed miRNAs between CRC cell lines. Venn diagrams show the overlap of differentially expressed miRNAs identified in Caco-2 and HCT116 cells relative to HCoEpiC. Differentially expressed miRNAs were defined as those with an adjusted *p*-value < 0.05 and |log_2_FC| > 1. (**A**) All differentially expressed miRNAs. (**B**) Upregulated miRNAs. (**C**) Downregulated miRNAs. Numbers indicate miRNAs unique to each cell line or shared between both cell lines. The overlap in panel (**A**) includes miRNAs with both concordant and opposite directions of change, whereas the overlaps in panels (**B**,**C**) include only miRNAs changing in the same direction in both CRC cell lines.

Differential expression analysis revealed extensive miRNA dysregulation in CRC cells compared with normal epithelial cells. In Caco-2 cells, 460 miRNAs were differentially expressed relative to HCoEpiC, including 277 upregulated and 183 downregulated miRNAs. In HCT116 cells, 541 miRNAs were differentially expressed, including 352 upregulated and 189 downregulated miRNAs. To identify miRNAs consistently dysregulated across CRC cell lines, the overlap between differentially expressed miRNAs in Caco-2 and HCT116 cells relative to HCoEpiC was assessed. A total of 299 miRNAs were differentially expressed in both CRC cell lines, whereas 161 and 242 miRNAs were uniquely differentially expressed in Caco-2 and HCT116 cells, respectively (Figure 2A). Among the shared differentially expressed miRNAs, 222 showed concordant directions of change: 146 were upregulated in both cell lines (Figure 2B) and 76 were downregulated in both cell lines (Figure 2C). The remaining 77 miRNAs showed opposite directions of change between the two CRC cell lines. Among the 29 prioritized cellular candidates, supplementary interaction analysis identified significant cell line × collection-time interactions for miR-205-5p and miR-30c-2-3p (FDR = 0.0150 and 0.0312, respectively).

**Figure 3:**
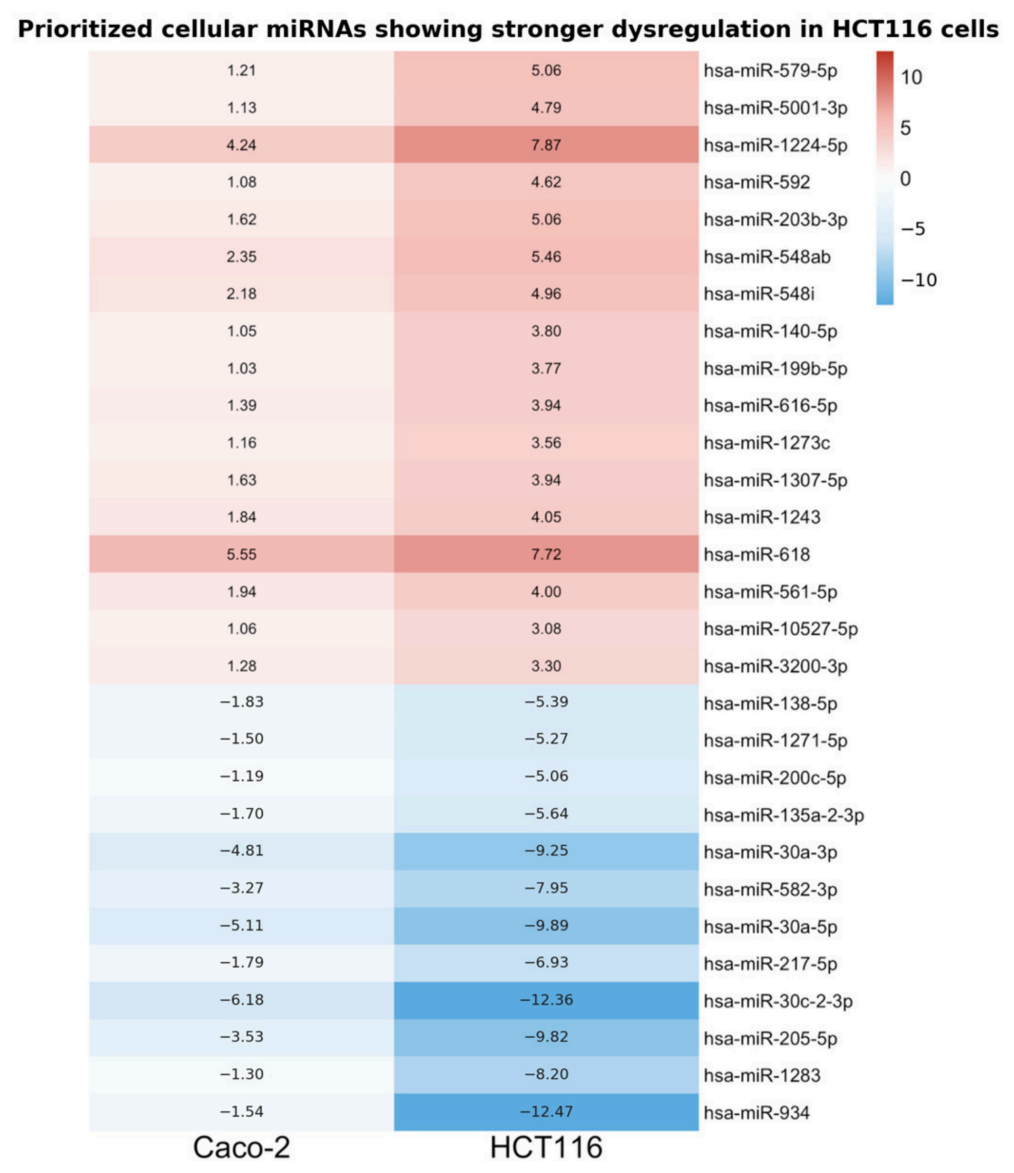
Prioritized cellular miRNAs showing stronger dysregulation in HCT116 cells. Heatmap of prioritized common differentially expressed miRNAs identified in Caco-2 and HCT116 cells compared with HCoEpiC controls. Rows are ordered according to Δlog_2_FC, whereas heatmap colors and displayed values represent the actual log_2_FC of each miRNA in Caco-2 and HCT116 cells relative to HCoEpiC cells.

To prioritize shared alterations with greater effect sizes in HCT116 cells, subsequent analyses focused on miRNAs showing concordant changes in both CRC models but stronger dysregulation in HCT116 than in Caco-2 cells. Among the 146 miRNAs upregulated and 76 miRNAs downregulated in both CRC cell lines, 66 upregulated and 48 downregulated miRNAs met this criterion. Candidates were further prioritized using quartile-based thresholds of Δlog_2_FC, calculated as the log_2_FC in HCT116 minus the log_2_FC in Caco-2. Upregulated miRNAs in the upper quartile (highest 25%) and downregulated miRNAs in the lower quartile (lowest 25%) were retained. The corresponding Δlog_2_FC thresholds were 1.97 for upregulated miRNAs and −3.18 for downregulated miRNAs, yielding 17 upregulated and 12 downregulated candidates for further analysis. The resulting 29 prioritized candidates are shown in Figure 3. Additionally, time-specific model estimates retained the original direction of change for all 29 candidates in both CRC cell lines at 24, 48, and 72 h, indicating that the detected interactions reflected changes in effect magnitude without reversal of direction (Supplementary Figure S2).

### 2.2. Shared Cellular-EV miRNAs Show Concordant Dysregulation and Target Enrichment in CRC-Relevant Pathways

Global EV-associated miRNA expression and differential expression patterns are summarized in the Supplementary Results S3 and Supplementary Figures S6 and S7.

**Figure 4:**
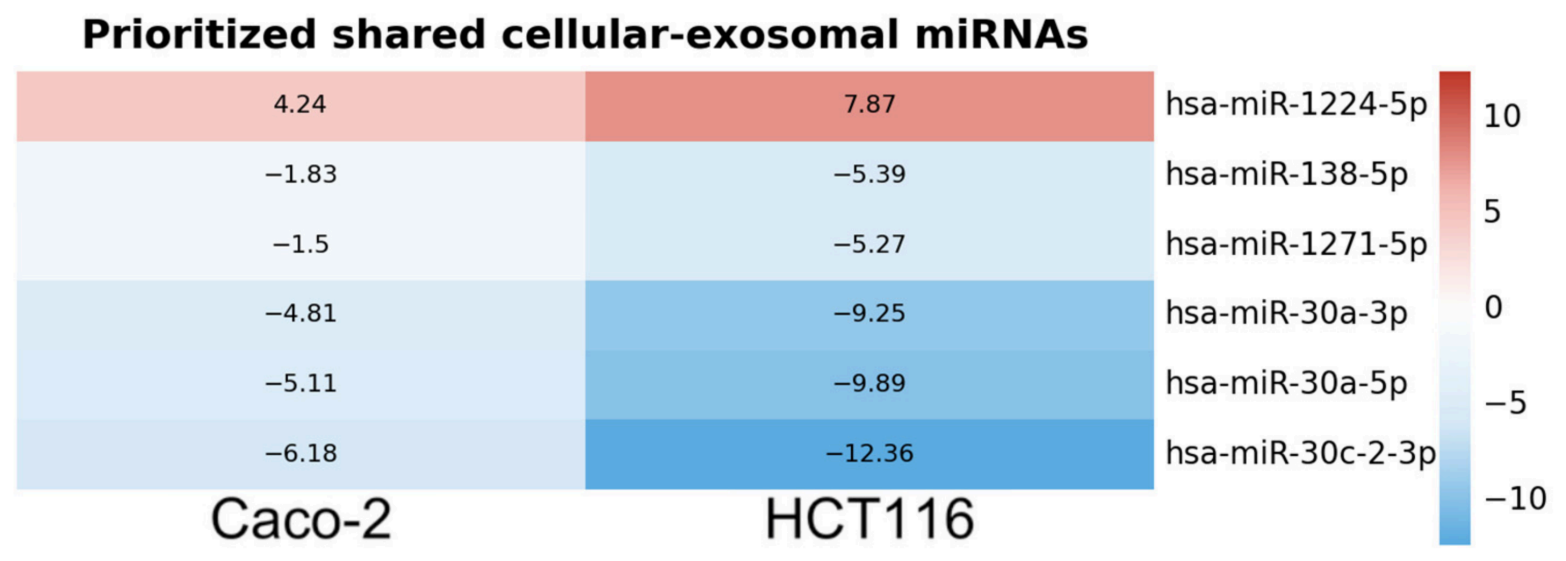
Prioritized miRNAs showing concordant dysregulation across cellular and EV compartments. Heatmap showing EV-associated log_2_FC values for the seven prioritized shared cellular-EV miRNAs in Caco-2- and HCT116-derived EVs relative to HCoEpiC-derived EVs. Rows are ordered by Δlog_2_FC, and heatmap colors and displayed values represent EV-associated log_2_FC values.

The 29 prioritized cellular miRNAs were subsequently evaluated in EVs from both CRC cell lines. Only miRNAs significantly altered in both EV datasets and showing the same direction of change as in the corresponding cellular compartment were retained. Candidates were further required to show greater absolute log_2_FC in HCT116-derived EVs than in Caco-2-derived EVs, with both calculated relative to HCoEpiC-derived EVs. This filtering yielded 7 shared cellular-EV miRNAs, including 2 upregulated and 5 downregulated (Figure 4). Among these seven candidates, significant cell line × collection-time interactions were detected in EVs for miR-30a-3p, miR-30a-5p, and miR-30c-2-3p (BH-adjusted FDR = 6.27 × 10^−7^, 2.27 × 10^−3^, and 2.27 × 10^−3^, respectively). All seven candidates retained their original model-estimated direction of change in both CRC cell lines relative to HCoEpiC-derived EVs at all three collection times (Supplementary Figure S3). Notably, three of the five downregulated miRNAs were mature strands originating from two miR-30 loci: miR-30a-3p and miR-30a-5p from MIR30A, and miR-30c-2-3p from MIR30C2.

**Figure 5:**
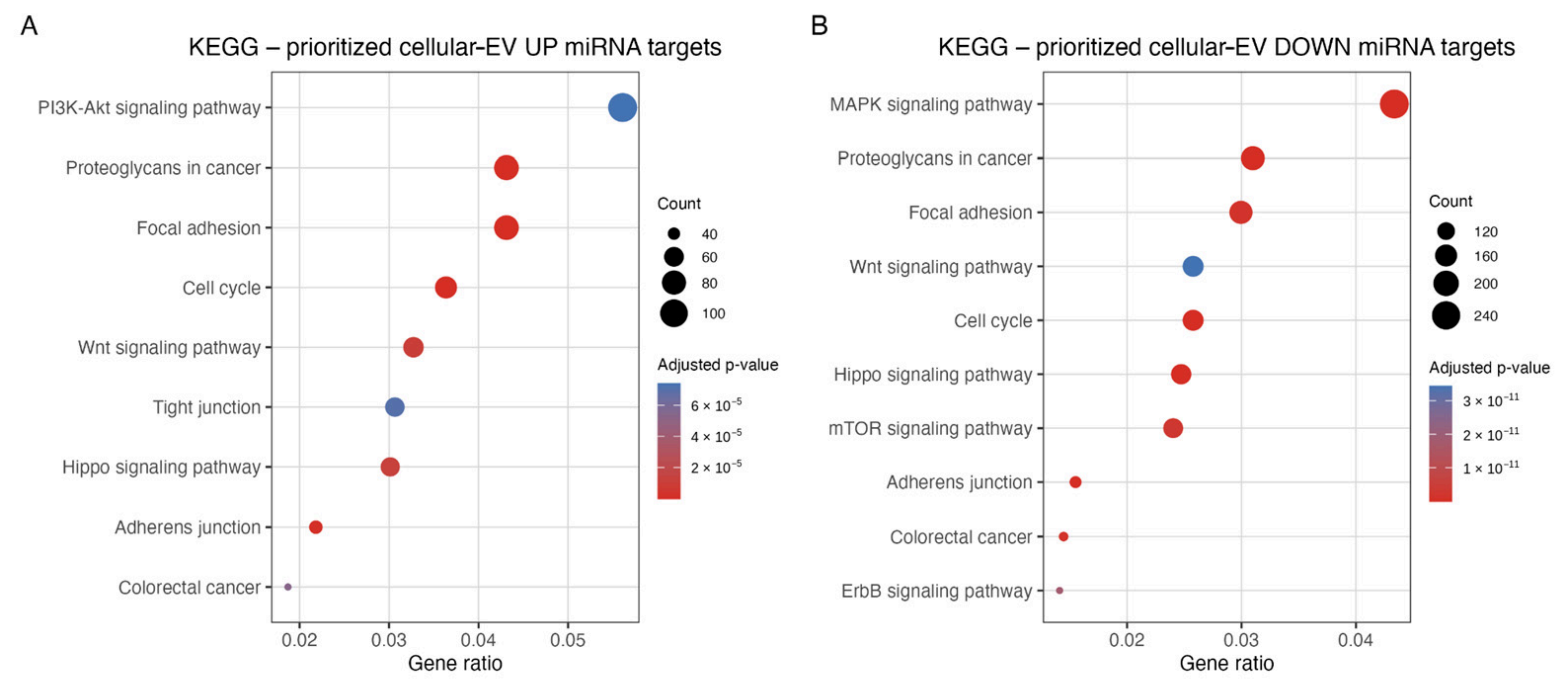
KEGG pathway enrichment of experimentally validated target genes of the seven shared cellular-EV miRNAs. (**A**) Nine selected cancer- and CRC-relevant pathways enriched among targets of the two upregulated miRNAs. (**B**) Ten selected cancer- and CRC-relevant pathways enriched among targets of the five downregulated miRNAs. The *x*-axis represents the gene ratio, dot size indicates the number of target genes associated with each pathway, and color represents the Benjamini–Hochberg-adjusted *p*-value. Pathways are ordered by gene ratio. Complete significant enrichment results are provided in Supplementary Data S1.

To provide biological context for these seven shared cellular-EV miRNAs, KEGG pathway enrichment analysis was performed separately on experimentally validated target genes of the upregulated and downregulated groups. Both target-gene groups showed enrichment in CRC-relevant pathways, including Wnt and Hippo signaling, cell cycle, and focal adhesion (Figure 5). Complete significant enrichment results are provided in Supplementary Data S1.

### 2.3. Patient Tissues Reproduce a Subset of the miRNA Alterations Identified in CRC Cell Models

**Figure 6:**
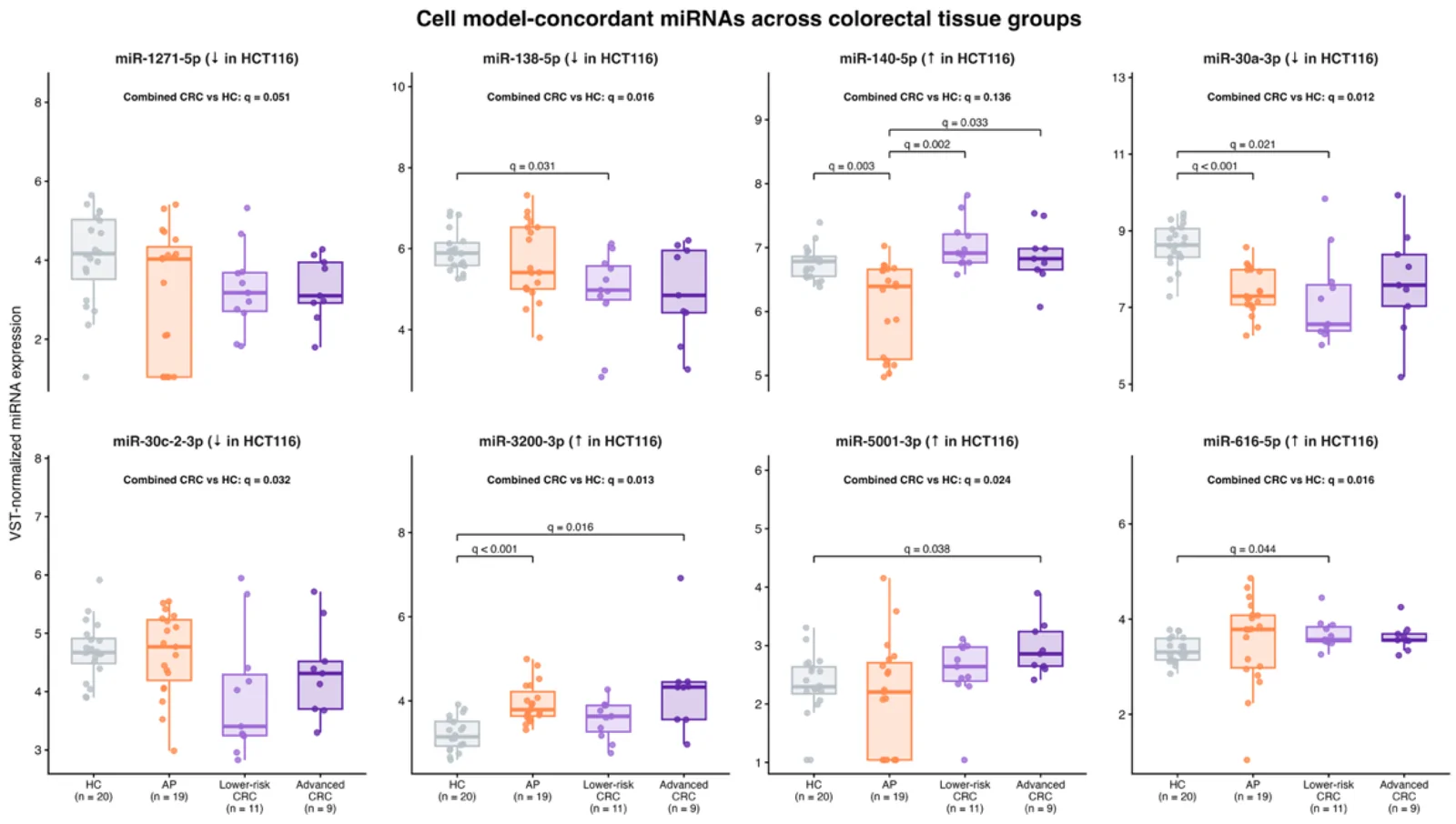
Expression of clinically concordant miRNAs across colorectal tissue groups. VST-normalized expression of eight miRNAs showing FDR-significant differences between CRC and HC tissues, with the direction of change consistent with that observed in both CRC cell lines relative to HCoEpiC. Samples are shown separately for healthy colorectal tissue (HC), adenomatous polyps (AP), lower-risk CRC, and advanced CRC. Individual points represent tissue samples, and boxplots show the median and interquartile range. Upward (↑) and downward (↓) arrows indicate upregulation and downregulation, respectively, in both CRC cell lines relative to HCoEpiC. Pairwise comparisons were performed using two-sided Mann–Whitney U tests, with Benjamini–Hochberg correction applied separately across the 26 measurable candidate miRNAs for each contrast. Only pairwise comparisons that remained significant after FDR correction (q < 0.05) are shown.

Of the 29 miRNAs prioritized in CRC cell models, 26 were available for patient-tissue analysis. Eight miRNAs differed significantly between the combined CRC and HC groups after FDR correction, with the direction of change consistent with that observed in both CRC cell lines relative to HCoEpiC (Figure 6). miR-3200-3p, miR-616-5p, miR-140-5p, and miR-5001-3p were increased in CRC, whereas miR-138-5p, miR-30a-3p, miR-30c-2-3p, and miR-1271-5p were reduced (Supplementary Table S1). Of these eight miRNAs, five—miR-138-5p, miR-30a-3p, miR-30c-2-3p, miR-1271-5p, and miR-140-5p—were concordantly dysregulated across CRC cells, EVs, and patient CRC tissues. Notably, both miR-30 family members, miR-30a-3p and miR-30c-2-3p, were consistently reduced across all three systems. Network analysis of experimentally validated targets provided additional biological context for the clinically concordant miRNAs, highlighting CRC-relevant pathways (Supplementary Figure S8; Supplementary Table S2). Figure 6 shows CRC samples separately as lower-risk and advanced CRC to illustrate distribution across the predefined clinicopathological subgroups. However, none of the eight miRNAs differed significantly between these subgroups after FDR correction.

In addition, the examination of the adenoma group showed that miR-30a-3p expression was already reduced in AP compared with HC (FDR q = 0.00023), with no significant difference between AP and CRC (q = 0.895). In contrast, miR-30c-2-3p did not differ significantly between AP and HC (q = 0.833) or between AP and CRC (q = 0.141). Thus, these comparisons did not support a stepwise decrease in either miRNA across HC, AP, and CRC.

To assess tissue concordance beyond the prioritized subset, we additionally evaluated the complete set of 222 miRNAs concordantly dysregulated in both CRC cell lines relative to HCoEpiC. Of these, 205 were available in the tissue dataset, and 26 showed significant differences between CRC and HC in the same direction as in the cellular models (16 upregulated and 10 downregulated; BH FDR < 0.05, with correction applied across all 205 evaluated miRNAs; Supplementary Figure S9).

### 2.4. Independent Evaluation of Tissue-Concordant miRNAs in TCGA-COAD/READ

The eight miRNAs were further evaluated in TCGA-COAD/READ miRNA-sequencing data [12], including 605 primary CRC and 11 matched adjacent non-tumor samples. After multiple-testing correction, four miRNAs showed differential expression consistent with that observed in CRC cell models and patient tissues: miR-140-5p was upregulated, whereas miR-30a-3p, miR-30c-2-3p, and miR-1271-5p were downregulated in CRC (Supplementary Table S3). Notably, reduced expression of both miR-30 family candidates, miR-30a-3p and miR-30c-2-3p, was again independently reproduced in TCGA-COAD/READ. We also evaluated associations between the miRNAs and clinicopathological indicators of disease aggressiveness or overall survival. However, no significant associations were identified.

**Figure 7:**
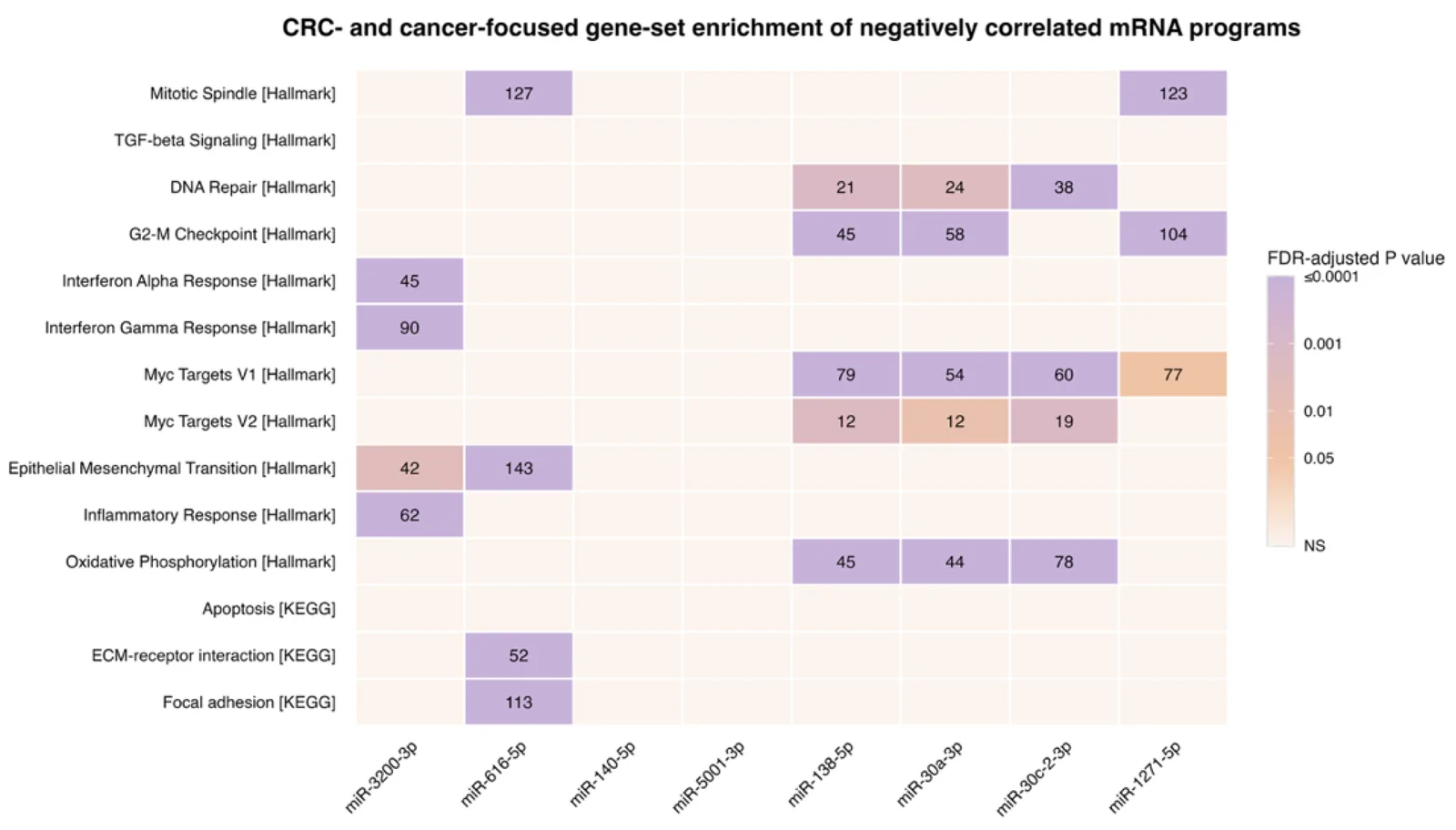
CRC- and cancer-focused gene-set enrichment of negatively correlated mRNA programs associated with the eight clinically concordant miRNAs in TCGA-COAD/READ. Gene-set enrichment was performed using genes showing consistent negative correlations with each miRNA in TCGA-COAD and TCGA-READ. Selected Hallmark and KEGG gene sets are shown. Numbers within cells indicate the number of overlapping genes, whereas color intensity represents FDR-adjusted enrichment significance. NS, not significant (FDR ≥ 0.05); values ≤ 0.0001 share the maximum color intensity.

Furthermore, integrated analysis of miRNA and mRNA expression data from the TCGA dataset revealed distinct tumor-associated transcriptional programs linked to individual miRNAs (Figure 7). Genes negatively correlated with miR-30a-3p, miR-1271-5p, miR-138-5p, and miR-616-5p were enriched in cell-cycle-related programs, particularly the G2/M checkpoint and mitotic spindle. MYC-target, DNA-repair, and oxidative-phosphorylation programs were also recurrently enriched, particularly in association with miR-30a-3p, miR-30c-2-3p, and miR-138-5p. These tumor-level correlations indicate associations with transcriptional programs rather than direct miRNA-mediated regulation.

## 3. Discussion

This study identified a subset of CRC-associated miRNA alterations showing concordant changes across cellular models, EVs, and human colorectal tissues. Of the 29 prioritized cellular miRNA candidates, seven met the EV selection criteria and retained the cellular direction of change. Five of these also showed concordant alterations in patient CRC tissues, and four were independently reproduced in TCGA. The partial overlap between cellular and EV-associated alterations may reflect both the applied selection criteria and selective miRNA sorting through mechanisms involving RNA-binding proteins and sequence motifs [10,11]. Cross-system evaluation narrowed the candidate set to recurrent alterations, among which reduced expression of miR-30a-3p and miR-30c-2-3p represented the most prominent family-level pattern. Further, we performed target-based pathway enrichment, as well as TCGA mRNA-miRNA correlation analysis, both of which indicated potential associations with proliferative, DNA-repair, and metabolic programs, including PI3K-Akt, focal adhesion, adherens junction, and p53 signaling. However, these analyses do not establish direct regulation in the studied systems, and correlations in whole-tumor expression profiles may partly reflect differences in malignant, stromal, and immune-cell composition [13].

Among the seven miRNAs concordantly dysregulated in the cellular and EV compartments, five were downregulated, including three mature miR-30 strands originating from two loci. miR-30a-3p and miR-30a-5p are derived from MIR30A, whereas miR-30c-2-3p is derived from MIR30C2. Their concordant downregulation therefore represents a pattern involving two miR-30 loci rather than three independent locus-level observations. Previous exploratory profiling identified reduced miR-30a-3p and miR-30a-5p abundance in serum-derived exosomes from CRC patients compared with healthy donors [14]. More recently, the CLEAR study included miR-30c-2-3p in a postoperative serum exosomal miRNA panel associated with CRC recurrence [15]. Our findings complement these reports by demonstrating concordant reductions in CRC cells and their released EVs. Two of these miRNAs, miR-30a-3p and miR-30c-2-3p, were also reduced in patient CRC tissues, and both alterations were independently reproduced in TCGA. The reduction in miR-30a-3p in AP suggests that this alteration may already be present before invasive carcinoma. However, its similar expression in AP and CRC, together with the absence of a significant miR-30c-2-3p change in AP, does not support a shared progressive decrease across the adenoma-carcinoma sequence. These cross-sectional comparisons cannot establish the timing of miRNA changes within individual patients. This recurrence across experimental compartments and independent tissue datasets strengthens their prioritization for further investigation, although it does not establish selective EV loading, functional transfer to recipient cells, or corresponding changes in circulating EVs from patients. The miR-30 family participates in diverse physiological processes, including cellular differentiation, autophagy, and metabolic regulation. Its members have been reported to exert both tumor-suppressive and oncogenic effects depending on tissue context and the targets involved, highlighting the need to interpret individual miR-30 alterations within the specific biological setting [16]. At the family level, reduced miR-30 activity has been linked to CRC progression, including enhanced proliferation, migration, invasion, and angiogenesis through impaired repression of the TCF7-MYC-cyclin D1 axis [17]. More recently, reduced miR-30a-5p expression was linked to 5-fluorouracil resistance, whereas its restoration increased treatment sensitivity by targeting LDHA-mediated glucose metabolism [18]. Additional functional evidence links miR-30a-5p to the suppression of CRC-cell migration and invasion through direct targeting of *IGF1R*, accompanied by reduced AKT phosphorylation and altered EMT-related protein expression [19]. Previous functional work also showed that inhibition of miR-30a-3p promoted CRC-cell growth, migration, and invasion through regulation of *SOAT1* [20]. Direct evidence linking miR-30c-2-3p to cell-cycle regulation has been reported in breast cancer cells, where it targeted *CCNE1* and *TRADD*, reducing cell-cycle progression, proliferation, and invasion [21]. This provides mechanistic context for our findings, although these interactions remain to be established in CRC.

Furthermore, mRNA-miRNA correlation analysis indicated that miR-30 candidates were associated with proliferative, DNA-repair, and metabolic transcriptional programs. Complementary functional evidence showed that miR-30a suppressed KRAS-mutant colorectal tumorigenesis by directly targeting *KRAS* and *ME1*, reducing NADPH production and lipid metabolism [22]. Together, these studies provide biological context for the recurrent miR-30 alterations identified here. Concordant reductions in miR-30a-3p and miR-30c-2-3p across experimental and clinical datasets strengthen their prioritization for mechanistic investigation, although they do not establish a common cellular origin or mechanism across systems. Importantly, membership of the same miRNA family does not imply interchangeable functions: mature 3p and 5p strands have distinct sequences and may regulate different targets. Evidence from miR-30a-5p or precursor-level studies therefore cannot be directly attributed to miR-30a-3p or miR-30c-2-3p. The observed pattern warrants functional characterization of each mature miRNA and its targets in relevant CRC models.

Other candidates provided additional evidence of cross-system concordance. miR-138-5p and miR-1271-5p were reduced across cells, EVs, and patient tissues, and the reduction in miR-1271-5p was also reproduced in TCGA. Both miR-138-5p and miR-1271-5p have been linked to tumor-suppressive functions in CRC, including the restriction of proliferation, migration, and invasion through distinct molecular mechanisms, with miR-138-5p additionally implicated in chemoresistance [23,24,25]. In contrast, miR-140-5p was consistently increased across experimental and clinical datasets, whereas previous studies reported reduced expression and tumor-suppressive activity through targets including *SOX4* and *VEGFA* [26,27]. This discrepancy may reflect differences in biological context, cohort composition, and analytical methodology across studies [28]. The other upregulated cellular-EV candidate, miR-1224-5p, has also been reported to suppress CRC-cell migration, invasion, and epithelial–mesenchymal transition through SP1-mediated NF-κB signaling [29]. However, increased miR-1224-5p abundance has been reported in serum exosomes from patients with CRC [30]. Together, these observations indicate that expression direction alone is insufficient to assign a tumor-promoting or tumor-suppressive role to these candidates. At the transcriptomic level, miRNA-mRNA correlation analysis associated miR-1271-5p with G2/M-checkpoint and mitotic-spindle programs, whereas no significant gene-set associations were detected for miR-140-5p after FDR correction. Because these analyses used whole-tumor expression profiles containing malignant, stromal, and immune-cell signals [13], the associations may partly reflect tissue composition and do not establish direct miRNA-mediated regulation.

Cross-system recurrence was not significantly associated with the evaluated clinical features. None of the eight clinically concordant miRNAs differed significantly between lower-risk and advanced CRC after FDR correction. No significant associations with clinicopathological indicators of disease aggressiveness or overall survival were identified in TCGA either. The small subgroup sizes and composite clinicopathological classification in the initial patient cohort limit interpretation, while molecular and microenvironmental heterogeneity may further obscure subgroup differences [13,31]. Thus, reproducibility of CRC-associated expression changes does not, by itself, establish their prognostic value. This study has several limitations. Candidate selection was restricted to two CRC cell models and prioritized larger absolute expression changes in HCT116, potentially excluding other reproducible alterations. These models represent only part of CRC molecular heterogeneity, requiring evaluation in additional models. Although serum supplementation was standardized to 5% exosome-depleted FBS during the collection period, differences in basal media, prior culture conditions, and proliferation rates may have influenced cellular and EV-associated miRNA profiles. The cell-line comparisons served as an initial discovery step, and concordant changes in patient tissues, including four alterations independently reproduced in TCGA-COAD/READ, support the relevance of selected candidates to human CRC. However, culture-related effects on candidate selection cannot be excluded.

The other methodological caveat was that precipitation-based isolation may have co-isolated lipoproteins and protein-RNA complexes that contributed to the recovered miRNA profiles. Detection of EV-associated markers and membrane-enclosed vesicles, together with ApoA1 below the assay’s detection range, does not establish an exclusively vesicular RNA origin. No nuclease protection assay was performed; therefore, these results represent EV-enriched preparations rather than exclusively intravesicular cargo. Furthermore, conditioned medium was not replaced over 24–72 h, and EV input was standardized by medium volume rather than donor-cell or particle number. The observed profiles may thus reflect differences in cell abundance, EV release, and extracellular RNA accumulation alongside changes in miRNA cargo.

Secondly, the patient cohort was small, particularly for clinical subgroup comparisons. Finally, database-derived target mapping and miRNA-mRNA correlations do not establish direct regulatory effects. Functional validation in additional CRC models and patient-derived systems remains necessary.

In conclusion, cross-system evaluation identified a reproducible subset of CRC-associated miRNA alterations. Five miRNAs—miR-138-5p, miR-30a-3p, miR-30c-2-3p, miR-1271-5p, and miR-140-5p—showed concordant changes across cells, EVs, and patient tissues, and four of these alterations were independently reproduced in the TCGA dataset. Within this subset, the consistent reduction in miR-30a-3p and miR-30c-2-3p highlighted a recurrent miR-30-family pattern across experimental and clinical datasets. These findings demonstrate the value of evaluating cell-model-derived miRNA candidates across biological systems to prioritize reproducible CRC-associated alterations for mechanistic investigation.

## 4. Materials and Methods

### 4.1. Cell Culture

Human colon adenocarcinoma cell lines HCT116 (CCL-247™, ATCC, Manassas, VA, USA) and Caco-2 (HTB-37™, ATCC, Manassas, VA, USA) were maintained in McCoy’s 5A medium (Sigma-Aldrich, St. Louis, MO, USA) supplemented with 10% fetal bovine serum (FBS; Gibco, Grand Island, NY, USA) and in Ham’s F-12K (Kaighn’s) medium (Gibco, Grand Island, NY, USA) supplemented with 20% FBS, respectively. Non-malignant human colonic epithelial cells (HCoEpiC, Innoprot, Derio, Spain) were cultured in colonic epithelial cell medium (Innoprot) containing 2% FBS and colonic epithelial cell growth supplement. Penicillin-streptomycin (1%; Gibco) was added to all culture media. Cells were maintained at 37 °C in a humidified atmosphere with 5% CO_2_. Subculture was performed using Accutase™ (Gibco). All cell lines were routinely tested and confirmed to be free of mycoplasma contamination.

### 4.2. Experimental Design

For the experimental model, Caco-2 and HCT116 cells were selected as biologically distinct CRC cell lines representing, respectively, a more differentiated enterocyte-like phenotype and a less differentiated phenotype with higher tumorigenic potential [32,33,34]. These models were selected to identify miRNA alterations shared across two biologically distinct CRC cellular phenotypes. Requiring concordant dysregulation in both CRC cell lines relative to HCoEpiC was intended to prioritize shared alterations over changes observed in only one model. For each cell type, cells were cultured in 75 cm^2^ culture flasks for cellular RNA isolation and conditioned-medium collection. Cultures were grown until they reached more than 70% confluence, which was defined as the experimental starting point. Subsequently, the routine culture medium was removed, and the cells were washed three times with phosphate-buffered saline (PBS). Fresh cell type-specific basal media supplemented with 5% exosome-depleted fetal bovine serum (FBS; Gibco, Grand Island, NY, USA) were then added to all three cell types. The medium was not replaced during the subsequent incubation period. Cells and conditioned media were collected from the culture flasks at the respective endpoints of 24, 48, or 72 h for cellular miRNA analysis and EV isolation, respectively, with two biological replicates per cell type at each time point. Cell line × collection-time interactions were additionally assessed in both datasets, with time-specific estimates for the 29 prioritized cellular and seven shared cellular-EV candidates presented in Supplementary Figures S2 and S3, respectively.

### 4.3. EV Isolation

Conditioned medium was collected and stored at −80 °C until EV isolation. After thawing, the medium was centrifuged at 2000× *g* for 30 min at 4 °C to remove cellular debris, filtered through a 0.22 μm membrane (Merck Millipore, Darmstadt, Germany), and incubated with Total Exosome Isolation reagent (Invitrogen, Carlsbad, CA, USA) at a conditioned medium-to-reagent ratio of 1:0.5 (*v*/*v*). Following incubation, the samples were centrifuged at 17,000× *g* for 1 h at 4 °C. The resulting EV pellets were resuspended in PBS and stored at −80 °C until downstream analysis. EV preparations were assessed using complementary molecular, morphological, and particle-based characterization approaches guided by the Minimal Information for Studies of Extracellular Vesicles 2023 (MISEV2023) recommendations [35]. Detailed characterization methods and results are provided in Supplementary Methods S1 and Supplementary Results S2, respectively (Supplementary Figures S4 and S5).

### 4.4. Total RNA Extraction and Small RNA Sequencing

Total RNA was isolated from cell samples using the miRNeasy Micro Kit (Qiagen, Hilden, Germany), while EV-associated RNA was extracted from EV-enriched preparations obtained from a total of 3.6 mL of conditioned medium per biological sample (two 1.8 mL aliquots) using the GenElute Single Cell RNA Purification Kit (Sigma-Aldrich), according to the manufacturer’s instructions. The conditioned-medium input volume was identical across all three cell types and collection time points. EV input was not additionally normalized to donor-cell number or particle concentration before RNA extraction. Cellular and EV-associated small RNA libraries were prepared using the Illumina TruSeq Small RNA Sample Preparation Kit (Illumina, San Diego, CA, USA) according to the manufacturer’s protocol, with at least 2 μg of total RNA used for cellular samples and at least 20 ng of RNA for EV-derived samples. Library preparation included 3′ and 5′ RNA adapter ligation, cDNA synthesis, polymerase chain reaction (PCR) amplification using sample-specific barcode sequences, and gel-based size selection. Library yield and quality were assessed using the Agilent 2100 Bioanalyzer (Agilent Technologies, Santa Clara, CA, USA). Cellular and EV-associated small RNA libraries were pooled separately and sequenced in independent runs on the Illumina HiSeq 2500 platform (Illumina), generating 1 × 50 bp single-end reads.

### 4.5. Bioinformatics and Statistical Analysis of Small RNA-Seq Data

Raw sequencing data were processed using nf-core/smrnaseq v1.0.0. Reads underwent quality control, adapter trimming, alignment to the GRCh37 reference genome, and miRNA annotation using miRBase v22.1; complete software details are provided in Supplementary Methods S2. Low-abundance miRNAs were removed, and differential expression was analyzed using DESeq2 v1.26.0 [36], with collection time included as a covariate. Differentially expressed miRNAs were defined by a Benjamini–Hochberg-adjusted *p*-value < 0.05 and |log_2_FC| > 1 [37]. PCoA was performed on variance-stabilized counts using Euclidean distances. For each miRNA concordantly dysregulated in both CRC cell lines relative to HCoEpiC, Δlog_2_FC was calculated as log_2_FC (HCT116 versus HCoEpiC) minus log_2_FC (Caco-2 versus HCoEpiC). This exploratory prioritization criterion was motivated by the contrasting differentiation and tumorigenic phenotypes of the two CRC models, together with the hypothesis that larger miRNA expression changes may have greater functional consequences. It was intended to focus follow-up analyses on shared alterations with larger effect sizes in HCT116. The resulting cellular candidates were subsequently evaluated in the corresponding EV datasets based on statistical significance, concordant direction of change, and greater absolute dysregulation in HCT116-derived than in Caco-2-derived EVs. Statistical analyses were performed using R v4.4.3 (R Foundation for Statistical Computing, Vienna, Austria).

### 4.6. miRNA–Target Interaction Analysis

Experimentally validated miRNA–target interactions were retrieved using multiMiR v1.28.0. KEGG pathway enrichment was performed with clusterProfiler v4.14.6 after conversion of gene symbols to Entrez Gene IDs. Benjamini–Hochberg-adjusted *p* < 0.05 defined significant enrichment. Detailed database, filtering, and visualization procedures are provided in Supplementary Methods S3.

### 4.7. Evaluation of Prioritized miRNAs in a Previously Published In-House Cohort

Colorectal tissue miRNA expression data from our previously published in-house cohort (GEO accession: GSE160432) were reanalyzed to evaluate the tissue relevance of the cell-model-derived miRNA pattern [38]. The tissue dataset was generated separately from the present cellular and EV datasets, using a different library preparation kit and sequencing instrument, with libraries prepared by a different researcher, and therefore represents a separately generated biological dataset. The analysis included batch-matched healthy colorectal tissues (HC, *n* = 20), adenomatous polyps (AP, *n* = 19), and CRC tissues (*n* = 20); the source cohort has been described previously [38]. For exploratory subgroup analysis, CRC cases with lymph-node involvement (N+), distant metastasis (M1), and/or poor differentiation (G3) were classified as advanced CRC (*n* = 9), and the remaining cases as lower-risk CRC (*n* = 11). Of the 29 prioritized cellular miRNAs, 26 were available in the patient dataset.

Variance-stabilized expression values were compared using Kruskal–Wallis and pairwise two-sided Mann–Whitney U tests. Lower-risk and advanced CRC were compared using a two-sided Mann–Whitney U test. Benjamini–Hochberg correction was applied separately across the 26 miRNAs for each comparison. Clinically concordant miRNAs were defined as those differing significantly between the combined CRC and HC groups after FDR correction and changing in the same direction in both CRC cell lines relative to HCoEpiC. These criteria identified the fixed eight-miRNA set subsequently evaluated in TCGA. For an additional exploratory analysis, we evaluated the complete set of 222 miRNAs concordantly dysregulated in both CRC cell lines relative to HCoEpiC, of which 205 were available in the tissue dataset. Variance-stabilized expression values were compared between the combined CRC and HC groups using two-sided Mann–Whitney U tests, with Benjamini–Hochberg correction applied across all 205 evaluated miRNAs. Tissue concordance required FDR < 0.05 and a difference in median expression in the same direction as the cellular changes.

### 4.8. Independent Evaluation of Tissue-Concordant miRNAs in TCGA-COAD/READ

Publicly available miRNA-sequencing and clinical data from the TCGA-COAD and TCGA-READ projects were obtained through the National Cancer Institute Genomic Data Commons [12,39]. The dataset comprised 605 primary CRC samples and 11 adjacent non-tumor samples, including 11 matched tumor–non-tumor pairs. The eight miRNAs identified in the patient-tissue analysis were evaluated individually. Differences between all CRC and adjacent non-tumor samples were assessed using two-sided Mann–Whitney U tests, whereas the matched subset was analyzed using paired Wilcoxon signed-rank tests. Benjamini–Hochberg correction was applied across the eight miRNAs. Associations with AJCC stage were evaluated using Spearman rank correlation and by comparing stages I–II with stages III–IV. Detailed data processing and statistical procedures are provided in Supplementary Methods S5.

### 4.9. Exploratory TCGA miRNA-mRNA Gene-Set Analysis

Matched miRNA and mRNA expression profiles from 603 primary tumors in the TCGA-COAD and TCGA-READ projects were used to investigate functional associations of the eight clinically concordant miRNAs. Spearman miRNA-mRNA correlations were calculated separately in the two projects and combined using a fixed-effect meta-analysis based on Fisher’s *z*-transformed correlation coefficients. Genes showing negative correlations in both projects and significant pooled associations after Benjamini–Hochberg correction were retained for over-representation analysis using a prespecified panel of MSigDB Hallmark and KEGG gene sets. Gene sets with an FDR q-value < 0.05 were considered significantly enriched. Detailed analytical procedures are provided in Supplementary Methods S6.

## Data Availability

The publicly available colorectal tissue miRNA dataset analyzed in this study is available in the Gene Expression Omnibus (GEO) under accession number GSE160432. TCGA-COAD and TCGA-READ data were obtained from the NCI Genomic Data Commons (GDC) Data Portal (https://portal.gdc.cancer.gov/ (accessed on 22 September 2026)). The newly generated small RNA-sequencing data have been deposited in the Gene Expression Omnibus (GEO) under accession number GSE353366.

## Supplementary Materials

The following supporting information can be downloaded at: https://www.mdpi.com/article/10.3390/ijms27219873/s1. References [1,2,3,4,40,41,42,43] are cited in the Supplementary Materials.
