# Supplementary material for: Cross-System miRNA Profiling Identifies a Clinically Recurrent miRNA Core in Colorectal Cancer
Source: Int J Mol Sci. 2026 Nov 4;27(21):9873. doi: 10.3390/ijms27219873 (PMC13764169; doi:10.3390/ijms27219873)
Supplement: Supplementary file 1 [file ijms-27-09873-s001.zip › Supplementary_Materials.pdf]

Supplementary methods

S1. Extracellular vesicle characterization

S1.1. Enzyme-linked immunosorbent assay

EV-associated proteins were assessed using sandwich enzyme-linked immunosorbent assay (ELISA) kits for CD63 (Invitrogen, Carlsbad, CA, USA, Cat. EH95RB), Glypican-1 (Invitrogen, Cat. EH222RB), HSP70 (Invitrogen, Cat. BMS2087), and ApoA1 (Invitrogen, Cat. EHAPOA1), according to the manufacturers' instructions. Optical density was measured at 450 nm with a 620 nm reference using a Tecan Sunrise microplate reader (Tecan Austria GmbH, Grödig, Austria). Assays were performed in duplicate.

S1.2. Nanoparticle tracking analysis

EV size distribution and particle concentration were assessed for Caco-2-derived EV preparations using a NanoSight NS300 equipped with a 405 nm laser (Malvern Instruments, Malvern, UK). EV suspensions were diluted 1:100 in PBS, and five 60 s videos were recorded per biological sample at 20-21 °C. Data were analyzed using NTA software v3.1 (Malvern Instruments, Malvern, UK), and mean values were calculated across the five recordings. NTA measurements were performed exclusively on Caco-2-derived EV preparations collected at 72 h. NTA measurements were available for Caco-2-derived preparations only and were therefore used as supplementary characterization rather than for quantitative comparison among cell lines.

S1.3. Cryo-transmission electron microscopy

Fresh EV suspensions were vitrified on glow-discharged holey carbon grids using a Vitrobot plunge-freezing device (FEI, Eindhoven, The Netherlands) and liquid ethane. Imaging was performed under liquid-nitrogen conditions using a 200 kV Glacios Cryo-TEM (Thermo Fisher Scientific, Eindhoven, The Netherlands) equipped with a direct electron detector and Volta Phase Plate.

S2. Additional small RNA-sequencing analyses

Raw reads were processed using nf-core/smrnaseq v1.0.0 with Nextflow v20.07.1 [40], Java v11.0.7, and Docker v19.03.12. FastQC v0.11.9, Trim Galore! v0.6.5, Bowtie v1.3.0 [41], miRBase v22.1 [42], the GRCh37 reference genome, mirtop v0.4.23, and MultiQC v1.9 [43] were used for quality control, adapter trimming, alignment, miRNA annotation, count generation, and reporting. Cellular and EV datasets were assessed separately based on the total number of miRNA-mapped reads and detected miRNAs. Outliers were identified from $\log_2$-transformed quality metrics using the 1.5 × interquartile-range criterion; two cellular samples were excluded, whereas all EV samples passed quality control. miRNAs with a mean raw count ≤ 1 were removed. Collection-time effects were evaluated using multidimensional scaling, and collection time was subsequently included as a covariate in the DESeq2 model. Differentially expressed miRNAs in both the cellular and EV datasets were defined by a Benjamini–Hochberg-adjusted p-value < 0.05 and $|\log_2 FC| > 1$. Cell line × collection-time interactions were assessed separately in the cellular and EV datasets using DESeq2 likelihood ratio tests comparing models with and without the interaction term, while accounting for culture blocks nested within cell line. Benjamini–Hochberg correction was applied separately across 852 cellular and 568 EV-associated miRNAs. Time-specific $\log_2$ fold changes relative to the corresponding HCoEpiC samples were examined

for the 29 prioritized cellular and seven shared cellular-EV candidates, with equal weighting across culture blocks.

S3. miRNA target and pathway analyses

Experimentally validated miRNA–target interactions were retrieved from miRecords, miRTarBase, and TarBase using the multiMiR R package v1.28.0. Gene symbols were converted to Entrez Gene identifiers using org.Hs.eg.db v3.20.0.

For the seven miRNAs concordantly dysregulated in the cellular and EV compartments, validated target genes were analyzed separately for the upregulated and downregulated miRNA groups. KEGG pathway enrichment was performed using the enrichKEGG function in clusterProfiler v4.14.6 with *Homo sapiens* specified as the target organism (organism = "hsa"). The default KEGG gene universe was used as the enrichment background, and pathways with a Benjamini–Hochberg-adjusted p-value < 0.05 were considered significant. Complete significant enrichment results are provided in Supplementary Data S1. Selected cancer- and CRC-relevant pathways are presented in Figure 5 of the main manuscript.

For the eight miRNAs showing directionally concordant alterations in CRC patient tissues, experimentally validated targets were subjected to KEGG enrichment and mapped to selected significantly enriched CRC-relevant pathways. The resulting miRNA-target-pathway network is shown in Supplementary Figure S8, and the corresponding significant KEGG enrichment results are provided in Supplementary Table S2.

S4. Evaluation of prioritized miRNAs in a previously published in-house cohort

Variance-stabilized expression values for the 26 measurable candidates in the GSE160432 dataset were compared among HC, AP, and CRC groups using Kruskal–Wallis tests followed by pairwise two-sided Mann–Whitney U tests. Lower-risk and advanced CRC subgroups were compared using a two-sided Mann–Whitney U test. Benjamini–Hochberg correction was applied separately across the 26 miRNAs for each comparison. Clinically concordant miRNAs were defined as those showing an FDR-significant difference between the combined CRC and HC groups and changing in the same direction in both CRC cell lines relative to HCoEpiC.

S5. TCGA validation of eight clinically concordant miRNAs

Mature miRNA expression was extracted from TCGA-COAD and TCGA-READ isoform-level files, expressed as $\log_2$(RPM + 1), and averaged across technical replicates. Differences between all primary CRC and adjacent non-tumor samples were assessed using two-sided Mann–Whitney U tests, whereas the 11 matched tumor-non-tumor pairs were analyzed using paired Wilcoxon signed-rank tests. For the matched analysis, expression change was defined as CRC relative to the corresponding adjacent non-tumor tissue. Benjamini–Hochberg correction was applied separately across the eight miRNAs within each analysis, with an FDR-adjusted p-value < 0.05 considered significant.

S6. Exploratory TCGA miRNA-mRNA gene-set analysis

Spearman correlations between each of the eight clinically concordant miRNAs and mRNA expression were calculated separately in TCGA-COAD (n = 443) and TCGA-READ (n = 160). The project-specific correlation coefficients were transformed using Fisher's z transformation and combined using a fixed-effect model weighted by the corresponding sample sizes ($n - 3$). Pooled estimates were transformed back to correlation coefficients, and two-sided p-values were calculated from the pooled Fisher z statistics. Benjamini–Hochberg correction was applied across the tested genes separately for each miRNA. Only genes showing negative correlations in both projects and a pooled FDR-adjusted p-value < 0.05 were retained for over-representation analysis. Enrichment was assessed using a prespecified panel of MSigDB v26.1.0 Hallmark and KEGG gene sets, with Benjamini–Hochberg correction applied across the tested gene sets. Gene sets with an FDR q-value < 0.05 were considered significantly enriched.

Supplementary results

S1. Collection-time assessment

No pronounced or systematic separation by collection time was observed in the cellular or EV datasets (Supplementary Figure S1). However, the absence of visible clustering does not exclude miRNA-specific temporal effects. Supplementary interaction analysis identified significant cell line × collection-time interactions for two of the 29 prioritized cellular candidates: miR-205-5p (FDR = 0.015) and miR-30c-2-3p (FDR = 0.031). Time-specific model estimates retained the original direction of change for all 29 candidates in both CRC cell lines at 24, 48, and 72 h (Supplementary Figure S2). Thus, the significant interactions reflected differences in effect magnitude without reversal of direction.

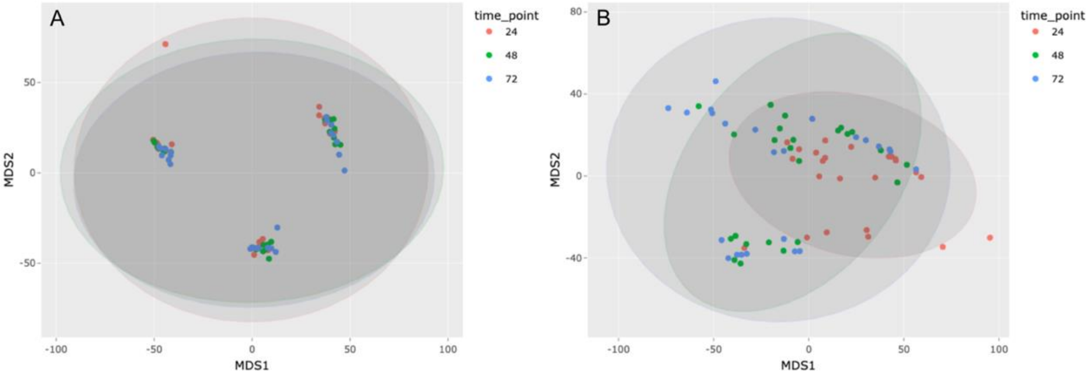

Supplementary Figure S1. Multidimensional scaling analysis according to collection time. (**A**) Cellular and (**B**) EV-associated miRNA profiles. Samples are colored by collection time (24, 48, or 72 h).

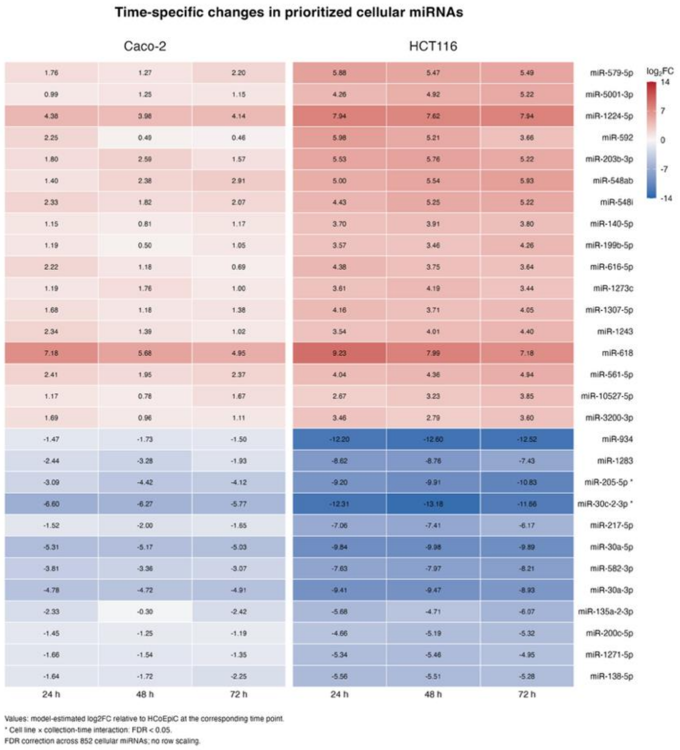

Supplementary Figure S2. Time-specific expression changes in the 29 prioritized cellular miRNAs. Heatmap showing model-estimated $\log_2$ fold changes in Caco-2 and HCT116 cells relative to HCoEpiC at 24, 48, and 72 h. Estimates were averaged with equal weight across culture blocks within each cell type. Colours and numerical values indicate $\log_2$ fold changes without row scaling. Asterisks denote significant cell line × collection-time interactions identified by likelihood ratio tests, with Benjamini–Hochberg correction across all 852 cellular miRNAs (adjusted $p < 0.05$). Asterisks do not indicate significance of individual time-specific comparisons.

Among the seven shared cellular–EV candidates, significant cell line × collection-time interactions were detected for miR-30a-3p, miR-30a-5p, and miR-30c-2-3p (BH-adjusted FDR = 6.27×10^{-7}, 2.27×10^{-3}, and 2.27×10^{-3}, respectively; correction across 568 EV-associated miRNAs). All seven candidates retained their original model-estimated direction of change in both CRC cell lines relative to HCoEpiC-derived EVs at 24, 48, and 72 h (Figure S3).

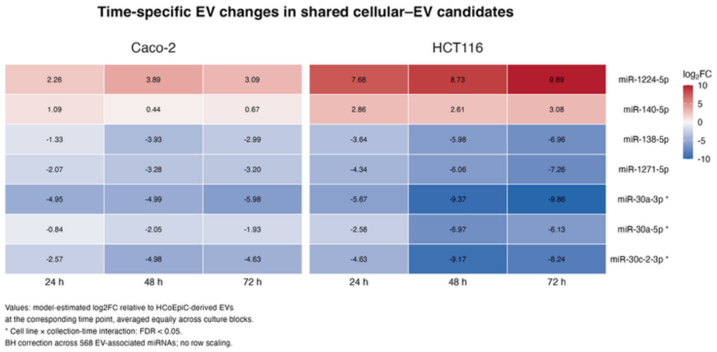

Figure S3. Time-specific dysregulation of the seven shared cellular-EV miRNA candidates in EV-enriched preparations. All seven candidates retained their original direction of change in both CRC cell lines at each collection time, despite significant cell line × collection-time interactions for three candidates.

S2. Extracellular vesicle characterization

ELISA confirmed EV-associated markers CD63, glypican-1, and HSP70 in EV preparations from all three cell lines, whereas ApoA1 levels were below the assay's detection range in the analyzed preparations. This finding does not exclude the presence of lipoproteins below the assay's detection range or other co-isolated non-vesicular components. Cryo-TEM showed rounded lipid-bilayer vesicles in preparations from all three cell lines (Supplementary Figure S4).

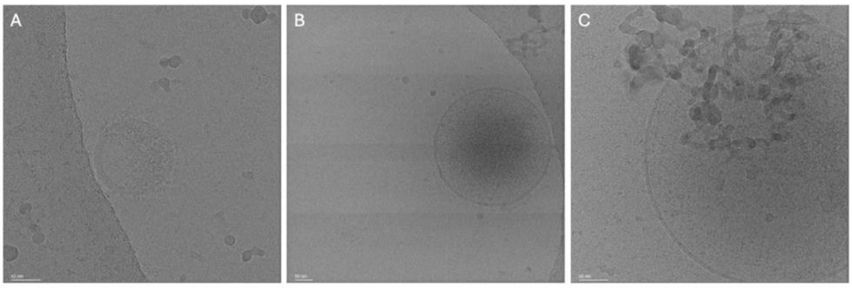

Supplementary Figure S4. Cryo-transmission electron microscopy characterization of extracellular vesicle preparations. Representative cryo-transmission electron microscopy images of EV preparations derived from (**A**) HCoEpiC, (**B**) Caco-2, and (**C**) HCT116 cells, showing membrane-enclosed particles with EV-compatible morphology. Scale bars, 50 nm.

NTA of EVs isolated from Caco-2-conditioned medium showed particle-size characteristics consistent with an EV-enriched preparation (Supplementary Figure S5). The mean particle diameter was 139.6 nm, the modal diameter was 104.7 nm, and the D50 was 124.4 nm. The mean reported particle concentration was 1.59×10^8 particles mL^{-1}. NTA measurements were available for Caco-2-derived EV preparations only and were therefore used as supplementary characterization rather than for quantitative comparison among the three cell lines.

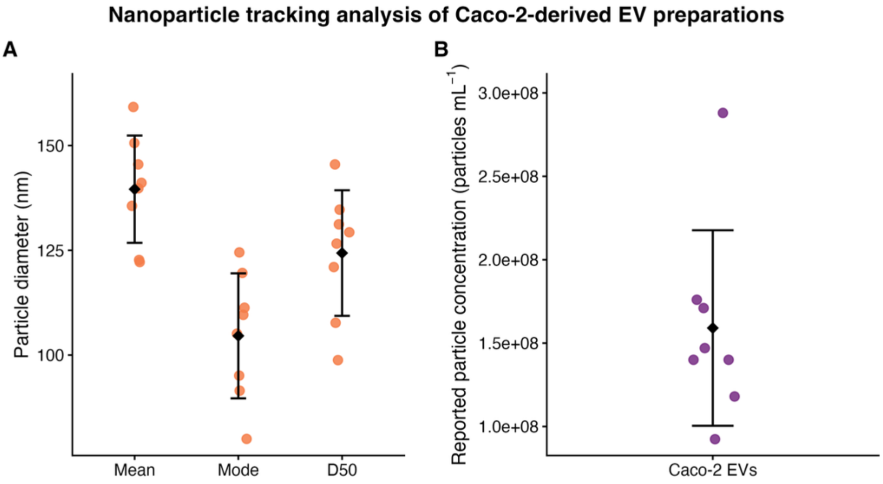

Supplementary Figure S5. Nanoparticle tracking analysis of Caco-2-derived extracellular vesicle preparations. EVs were isolated from Caco-2-conditioned medium collected at 72 h. **A** – Particle-size parameters, including mean diameter, mode diameter, and D50. **B** – Reported particle concentration. Colored points represent individual NTA reports (n = 8), black diamonds indicate the mean, and error bars represent the mean ± standard deviation.

S3. EV-associated miRNA expression

After quality control and filtering, 568 miRNAs were retained in the EV dataset. PCoA based on all retained miRNAs showed clear separation of EV samples according to their cell line of origin. Caco-2-derived EV samples formed a compact cluster distinct from HCoEpiC- and HCT116-derived EV samples, whereas HCT116-derived EV samples showed greater within-group dispersion. The first two

principal coordinates accounted for 32.8% and 17.1% of the variation, respectively, and together represented 49.9% of the variation captured by the analysis (Supplementary Figure S6).

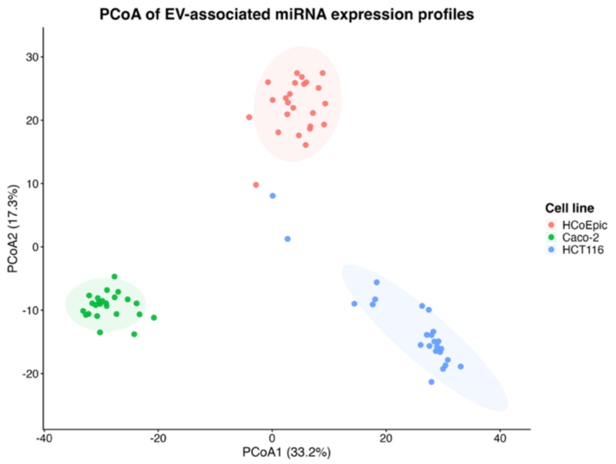

Supplementary Figure S6. Principal coordinate analysis of EV-associated miRNA expression profiles. PCoA was performed using Euclidean distances calculated from variance-stabilized expression values of all 568 miRNAs retained after quality control and filtering. Each point represents one EV sample, and colors indicate the cell line of origin. Shaded ellipses summarize the distribution of samples within each cell-line group at the 95% confidence level.

Differential expression analysis identified 246 miRNAs in Caco-2-derived EVs relative to HCoEpiC-derived EVs, including 147 upregulated and 99 downregulated miRNAs. In HCT116-derived EVs, 288 miRNAs were differentially expressed, including 200 upregulated and 88 downregulated miRNAs. A total of 143 differentially expressed miRNAs were shared between both CRC-derived EV datasets, whereas 103 and 145 were unique to Caco-2- and HCT116-derived EVs, respectively. Among the concordantly dysregulated miRNAs, 63 were upregulated, and 37 were downregulated in EVs from both CRC cell lines (Supplementary Figure S7).

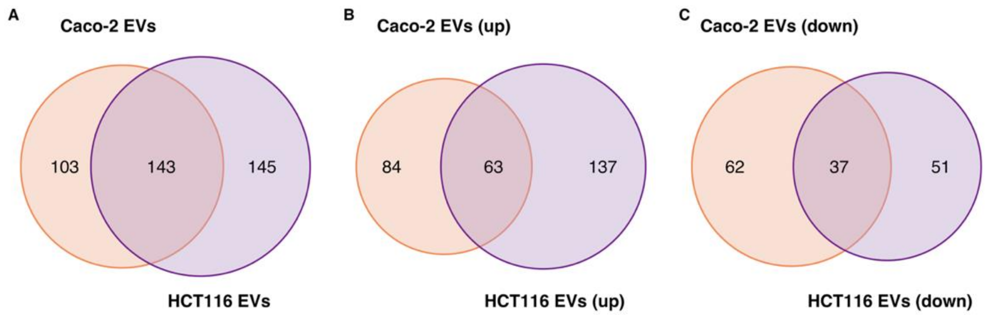

Supplementary Figure S7. Overlap of differentially expressed EV-associated miRNAs between CRC cell lines. Venn diagrams show the overlap of differentially expressed miRNAs identified in Caco-2- and HCT116-derived EVs relative to HCoEpiC-derived EVs. Differentially expressed miRNAs were defined as those with an adjusted p-value < 0.05 and $|\log_2 FC| > 1$. (**A**) All differentially expressed

miRNAs, (**B**) upregulated miRNAs, and (**C**) downregulated miRNAs. Numbers indicate miRNAs unique to each EV dataset or shared between both datasets.

S4. Clinically concordant miRNAs and validated targets

Eight miRNAs differed significantly between CRC and HC tissues after FDR correction, with the direction of change consistent with that observed in both CRC cell lines relative to HCoEpiC. Their cell-model-derived Δlog_2FC values and patient-tissue effect estimates are summarized in Supplementary Table S1. Analysis of experimentally validated targets linked five of these miRNAs to selected CRC-relevant pathways (Supplementary Figure S8). KEGG enrichment of the combined validated target set identified 25 significant pathways (Supplementary Table S2).

Supplementary Table S1. Cell-model and patient-tissue results for the eight clinically concordant miRNAs. Cell-model direction indicates concordant upregulation or downregulation in both CRC cell lines relative to HCoEpiC. Δlog_2FC was calculated as log_2FC(HCT116 versus HCoEpiC) minus log_2FC(Caco-2 versus HCoEpiC). Patient-tissue effects represent differences in median expression between CRC and HC tissues. FDR-adjusted *p*-values refer to the CRC-versus-HC comparison.

miRNA	Cell-model direction	Δlog_2FC	CRC-HC expression difference	FDR-adjusted *p-value*
hsa-miR-5001-3p	Upregulated	3.66	0.280	0.0357
hsa-miR-140-5p	Upregulated	2.75	0.253	0.0244
hsa-miR-616-5p	Upregulated	2.55	0.252	0.0054
hsa-miR-3200-3p	Upregulated	2.02	0.648	0.0054
hsa-miR-138-5p	Downregulated	–3.56	-0.791	0.0058
hsa-miR-1271-5p	Downregulated	–3.77	-0.881	0.0483
hsa-miR-30a-3p	Downregulated	–4.44	-1.226	0.0064
hsa-miR-30c-2-3p	Downregulated	–6.18	-0.535	0.0357

To examine the functional context of these clinically concordant miRNAs, experimentally validated targets were mapped to selected significantly enriched CRC-relevant pathways. Five of the eight miRNAs were linked to at least one selected pathway, with *EP300* representing the only validated target shared by two input miRNAs, miR-140-5p and miR-30a-3p (Supplementary Figure S8). miR-616-5p and miR-1271-5p had validated targets but were not linked to the selected pathways, whereas no validated targets were available for miR-5001-3p in the conservatively filtered interaction dataset. KEGG enrichment of the combined validated target set identified 25 significant pathways (Supplementary Table S2).

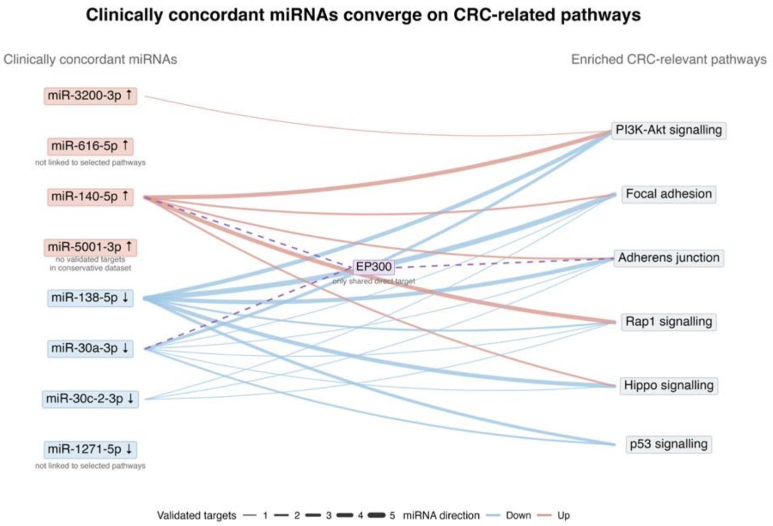

Supplementary Figure S8. Experimentally validated target genes linking clinically concordant miRNAs to selected CRC-relevant pathways. Solid connections link miRNAs to pathways through experimentally validated target genes, with line width indicating the number of targets supporting each miRNA-pathway connection. Dashed connections highlight *EP300*, the only validated target shared by more than one input miRNA.

Supplementary Table S2. KEGG pathway enrichment of experimentally validated targets of the eight clinically concordant miRNAs. Gene ratio represents the number of experimentally validated target genes assigned to a given pathway relative to the 78 mapped target genes included in the enrichment analysis. *P*-values were adjusted for multiple testing using the Benjamini–Hochberg method. Only pathways with a BH-adjusted *p*-value < 0.05 are shown.

KEGG ID	Pathway	Gene ratio	BH-adjusted P
hsa05206	MicroRNAs in cancer	15/78	9.02×10^{-6}
hsa04518	Integrin signaling	9/78	4.38×10^{-4}
hsa04520	Adherens junction	7/78	6.90×10^{-4}
hsa05218	Melanoma	6/78	0.0012
hsa04151	PI3K-Akt signaling pathway	12/78	0.0012
hsa04510	Focal adhesion	9/78	0.0012
hsa05205	Proteoglycans in cancer	9/78	0.0012
hsa05203	Viral carcinogenesis	8/78	0.0063
hsa04390	Hippo signaling pathway	7/78	0.0063
hsa04015	Rap1 signaling pathway	8/78	0.0063
hsa04115	p53 signaling pathway	5/78	0.0063
hsa05165	Human papillomavirus infection	10/78	0.0063
hsa04519	Cadherin signaling	11/78	0.0067
hsa05202	Transcriptional misregulation in cancer	7/78	0.0181
hsa04148	Efferocytosis	6/78	0.0244
hsa04066	HIF-1 signaling pathway	5/78	0.0253
hsa05163	Human cytomegalovirus infection	7/78	0.0298
hsa05164	Influenza A	6/78	0.0321

hsa04010	MAPK signaling pathway	8/78	0.0334
hsa05212	Pancreatic cancer	4/78	0.0355
hsa01521	EGFR tyrosine kinase inhibitor resistance	4/78	0.0388
hsa05219	Bladder cancer	3/78	0.0403
hsa03083	Polycomb repressive complex	4/78	0.0404
hsa05167	Kaposi sarcoma-associated herpesvirus infection	6/78	0.0443
hsa04371	Apelin signaling pathway	5/78	0.0456

S5. Directional concordance in matched TCGA tissues

The eight clinically concordant miRNAs were evaluated in 11 matched TCGA CRC and adjacent non-tumor tissue pairs. Four miRNAs – miR-140-5p, miR-30a-3p, miR-30c-2-3p, and miR-1271-5p – showed FDR-significant changes in the expected direction. miR-616-5p changed in the expected direction but was not significant after FDR correction, whereas miR-3200-3p changed significantly in the opposite direction. Neither miR-5001-3p nor miR-138-5p showed a statistically significant paired expression difference (Supplementary Table S3).

Supplementary Table S3. Directional concordance of eight clinically concordant miRNAs in matched TCGA CRC and adjacent non-tumor tissues.

**miRNA**	**Expected direction**	**Paired $\log_2$ change**	**FDR q**	**Concordance**
miR-3200-3p	↑	−1.666	0.0026	No
miR-616-5p	↑	+0.734	0.3661	Yes, not significant
miR-140-5p	↑	+3.738	0.0026	Yes
miR-5001-3p	↑	0.000	0.1667	Not established
miR-138-5p	↓	0.000	0.6875	Not established
miR-30a-3p	↓	−1.374	0.0219	Yes
miR-30c-2-3p	↓	−3.260	0.0026	Yes
miR-1271-5p	↓	−0.760	0.0039	Yes

S6. Tissue concordance of the broader cellular miRNA candidate set

To assess tissue concordance beyond the prioritized subset, we evaluated the complete set of 222 miRNAs concordantly dysregulated in both CRC cell lines relative to HCoEpiC. Of these, 205 were available in the patient-tissue dataset. Twenty-six miRNAs showed significant differences between CRC and HC tissues in the same direction as in the cellular models, including 16 upregulated and 10 downregulated miRNAs (Supplementary Figure S9). Statistical significance was defined as Benjamini–Hochberg FDR < 0.05, with correction applied across all 205 evaluated miRNAs.

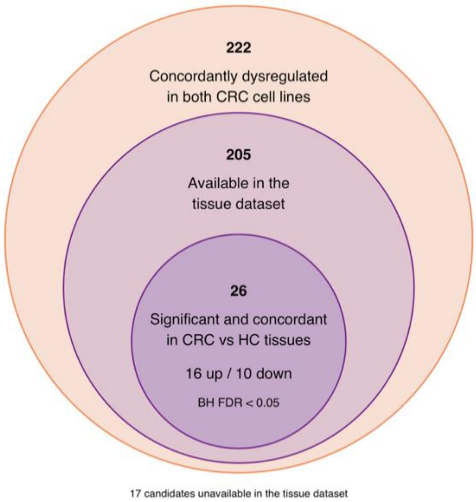

Supplementary Figure S9. Tissue concordance of the broader cellular miRNA candidate set. Nested circles illustrate the relationship between the complete cellular candidate set, candidates available for tissue analysis, and those meeting the tissue significance and directional-concordance criteria. Circle areas are schematic and not proportional to set sizes.

Supplementary materials

1. **Supplementary Data S1:** Complete significant KEGG pathway enrichment results for experimentally validated targets of prioritized shared cellular-EV miRNAs.
